# Can Sled-Resisted Priming Training Enhance Performance in Amateur Football Players?

**DOI:** 10.3390/s25216575

**Published:** 2025-10-25

**Authors:** Alvaro Medina-Sanchez, Jose Luis Felipe, Jorge Garcia-Unanue, Leonor Gallardo, Antonio Alonso-Callejo

**Affiliations:** 1 IGOID Research Group, Department of Physical Activity and Sport Sciences, University of Castilla-La Mancha, 45071 Toledo, Spain; alvaro.medina@uclm.es (A.M.-S.); jorge.garciaunanue@uclm.es (J.G.-U.); leonor.gallardo@uclm.es (L.G.); or antonio.alonso@universidadeuropea.es (A.A.-C.); 2 Performance Analysis Department, UD Las Palmas, 35019 Las Palmas de Gran Canaria, Spain; 3 Department of Sports Sciences, Faculty of Medicine, Health and Sports, Universidad Europea de Madrid, 28670 Madrid, Spain

**Keywords:** CMJ, sprint, F-V profile, soccer

## Abstract

(1) Background: This study examined the acute and chronic effects of sled-resisted training on power, strength, jump height (evaluated through a CMJ), and sprint kinematics in Spanish amateur football players. (2) Methods: A total of 19 football players (28 ± 8 years; 172 ± 12 cm; 79.3 ± 22.3 kg) performed 10 sled-resisted training sessions with loads of 80% (±2 kg) of their body mass on the day before the match (MD-1). Performance was assessed using a CMJ measured with the My Jump Lab app and sprint kinematics recorded with the 1080 Sprint system; both of these sensor-based technologies are validated for sports performance analysis. The CMJ was performed on MD-1 before the training and again on match day. Sprinting was evaluated before the sled-resisted training and again one week after it ended. (3) Results: These results suggest that sled training with heavy loads and low volume may not have acute effects on CMJ in amateur football players 24 h after it is performed, but there are chronic improvements in CMJ relative force and changes in sprinting after 10 weeks of sled-resisted training. (4) Conclusions: The findings of this study will enable football coaches and physical trainers to understand the changes in this training method to improve performance in amateur football players, using only three repetitions per player per week.

## 1. Introduction

In a football match, players run at different intensities, jump, dribble, and make contact with opponents [1]. Recent studies [2,3,4] have examined the evolution of physical demands in elite football leagues over recent seasons, finding that professional football players have increased their high-intensity running distance and the number of high-intensity efforts, but decreased the total distance covered.

In order to compete at peak performance, strategies such as post-activation potentiation (PAP), post-activation performance enhancement (PAPE), and priming are being studied [5]. PAP has been characterized as a physiological phenomenon with a short window of action (less than 4 min), whereas PAPE becomes evident after a few minutes, with a longer window of action than PAP (between 4 and 15 min) [6]. The delayed potentiation effect or priming is a training strategy performed 6–48 h before competition [7] with the aim of improving neuromuscular preparation and performance in athletes [5]. Research shows that priming exercise improves several measures of neuromuscular performance within 48 h [7,8].

Due to the increased physical demands in elite football in recent years [2], it has become necessary to regularly assess neuromuscular condition [9]. Thus, the countermovement jump (CMJ) has been one of the most widely used tests to determine lower limb neuromuscular performance in both individual and team sports [10], especially in football [11].

Furthermore, several studies have suggested the use of the horizontal force–velocity (F-V) profile to better understand sprinting and power skills in football [12]. The F-V profile is a two-axis linear regression (X-axis = velocity and Y-axis = force) developed by plotting the force applied at each velocity, as the higher the velocity, the lower the force that can be applied [13,14].

Despite the knowledge derived from existing research on the effects of priming interventions on CMJ and sprint performance [15,16], there remains a gap regarding the impacts of sled-resisted priming training on amateur football players. Therefore, the aim of this study was to examine the acute and chronic effects of the priming training method (sled-resisted training) on power, strength, and jump height performance, as well as sprint kinematics variables in Spanish amateur football players.

## 2. Materials and Methods

A total of 19 male football players (28 ± 8 years; 172 ± 12 cm; 79.3 ± 22.3 kg) from a Spanish amateur regional division team agreed to participate in this study. All participants were outfield players (defenders, midfielders, and forwards), with at least 3 years of regular football training experience. Inclusion criteria required players to be free of injury, actively competing during the season, and available for all testing sessions. Exclusion criteria included goalkeepers, recent injuries, or inconsistent attendance.

CMJ was measured with the My Jump Lab app (Carlos Balsalobre, Madrid, Spain), a valid and reliable instrument to measure jumping performance in real time and to evaluate CMJ height [17].

The 1080 Sprint (1080 Motion, Lidingö, Sweden) was used to record the sprints and to obtain the F-V profile. This linear encoder has a sampling frequency of 333 Hz [18] and provides kinematics measurements throughout the sprint. It is also valid for measuring the F-V profile in sprint acceleration [19]. Variables included for the analysis are presented in Table 1.

Both My Jump Lab and 1080 Sprint are sensor-based systems that allow accurate and real-time measurement of neuromuscular and sprint performance, respectively. Their use aligns with the scope of the journal, which focuses on sensor technologies applied to sports performance.

**Figure 1:**
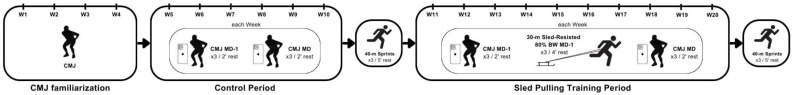
Timeline of the study procedure. W = week; BW = body weight.

Figure 1 represents the timeline of the study procedure. CMJ familiarization training was conducted without measurement in the 4 weeks prior to the start of the procedure. The research lasted 18 weeks (5 January–5 May 2024), but only the 16 competition weeks were evaluated (non-competition weeks were excluded).

The procedure consists of performing 3 CMJs. The CMJ protocol was as follows: the participant was instructed to stand comfortably with hands on hips, on signal, with bent knees, hips and ankles keeping their hands on hips and jumping vertically without pausing. No preparatory step or additional jump was allowed [20]. Each player performed 3 jumps (with 2 min of recovery between attempts). The best of the 3 jumps was chosen for statistical analysis. The CMJs were performed one day before the match (MD-1), and other CMJs on Match Day (MD). All jumps were performed always wearing the same sneakers, with no warm-up or activation beforehand. The video of each of the players jumping was recorded with the slow motion of an iPhone 11 Pro at 240 fps, placed two meters from the subject and positioned at ground level to capture the moment of take-off and landing of the first foot. Subsequently, the jump was measured by importing the video into the My Jump Lab application. Jumps were performed on the MD-1 and MD for 16 weeks. The control period was carried out during the first 6 weeks (5 January–18 February 2024).

Sled pulling training was performed for 10 weeks thereafter (23 February–3 May). Each player performed 3 weighted 30 m sprints with 80% (±2 kg) of their body weight, with a 4 min rest between sprints. Prior to sled-resisted sprint sessions, players completed a standardized warm-up based on the FIFA 11+ program [21]. The training was conducted on flat artificial turf under stable environmental conditions (temperatures between 15 and 20 °C, no rain or wind). A 15 kg sled (Iberian Sportech^®^, Sevilla, Spain) was used, and additional Iberian Sportech^®^ weight plates were added to reach the target load of 80% of each player’s body mass. The towing system consisted of a waist harness with a low attachment point at hip height to ensure horizontal force application. All sprints were initiated from a standing start, without blocks or preparatory steps, following a standardized verbal countdown. The surface was dry and consistent throughout the intervention period, and no slope or incline was present.

The measures with the 1080 Sprint were performed the day before the first day of priming training (22 February 2024) and the week after the last weekend of competition (9 May 2024). On both days, a warm-up was conducted according to the required intensity and then each player performed 3 × 40 m sprints at maximal speed with a 5 min rest between sprints. The fastest sprint was included in the analysis.

A linear mixed model was performed to evaluate the acute effects of sled training on CMJ variables, with one model for each dependent variable. The model included as independent variables with fixed effects the ‘MD-1–MD’ and the ‘study phase’ (sled = yes or sled = no). The variable ‘Player’, which identifies each player, was included as a random effect. The interaction of the variable Player with the variable Toma was also included as a random effect to analyze the time taken individually.

To analyze the differences between pre and post in sled training in the 1080 variables, a normality and homogeneity of variances analysis (Shapiro–Wilk test and Levene’s test, respectively) was applied. When normal distributions and homogeneity of variance were found, a paired samples Student’s T-test with a 95% confidence interval was used. All analyses were conducted using the statistical software R version 4.0.3 [22]. Effect sizes (ES) were calculated and interpreted according to Cohen’s thresholds: small (≥0.2), moderate (≥0.5), and large (≥0.8) [23].

## 3. Results

In the statistical analysis of the mixed model, significant values were found for relative force (0.1 N/kg; SE 0.04; T = 2.44; *p* < 0.05) for sled training compared to non-sled training (Table 2), which is considered long-term (chronic) improvement. No significant improvements were found for any of the remaining variables, considered as variables on acute performance improvements.

**Figure 2:**
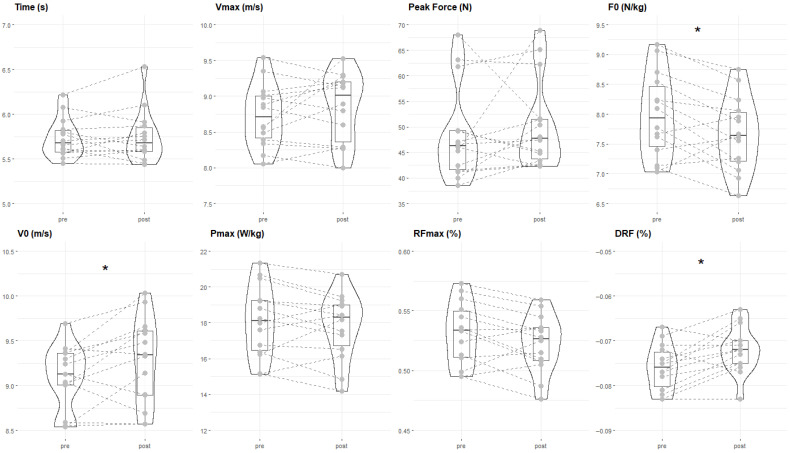
Comparison of pre (before performing the resisted training) and post (after performing the resisted training) sprint variables. Bars represent mean ± standard deviation (SD) and enhance the visual clarity. Time (s), V_max_ (m/s), Peak force (N). F-V sprint profile variables: F_0_ (N/kg), V_0_ (m/s), P_max_ (W/kg), RF_max_ (%), DRF (%). * Significant differences (*p* < 0.05).

In the statistical analysis of the paired samples t-test (Table 3), F_0_ was significantly lower post-intervention than pre-intervention (−0.31 N/kg; 95% CI −0.58 to −0.05; *p* < 0.05; ES = −0.47) (Figure 2). In contrast, V_0_ was significantly higher in post than in pre (0.22 m/s; 95% CI 0.03 to 0.4; *p* < 0.05; ES = 0.49) (Figure 2), as was DRF (0.01%; 95% CI 0.001 to 0.01; *p* < 0.05; ES = 0.77) (Figure 2). There were no significant differences in the rest of the variables.

## 4. Discussion

There are no studies that have evaluated the effects of priming training on sprinting and jumping performance in amateur football players. Existing studies focus on professional or semi-professional players. Therefore, one of the strengths of this study is its contribution to understanding the effects of this type of training on a broader population of football players.

This study analyzed the acute and chronic effects on power, strength, and jump height performance of a priming training regimen based on resisted sprint with 80% of body weight, performed 24 h before competition. Additionally, it examined the chronic effects on sprint kinematic variables. Few studies have investigated the effects of a high-load sled priming training session on sprint and CMJ performance in amateur football players, making the discussion of results more challenging [24,25].

The main finding of the study was that CMJ height, relative strength, and power did not significantly improve in the MD versus MD-1 period during the 10 weeks of priming training, compared to the difference between MD and MD-1 during the first 6 weeks without priming. This indicates that priming training did not produce a marked improvement in performance during its application. However, there was a longer-term improvement, as significant increases in relative strength were observed when comparing the sled-resisted training period with the non-sled-resisted training period [26]. Regarding sprint kinematic variables, significant differences were found in the sprint force–velocity sprint profile. Specifically, the variables V_0_ and DRF improved significantly, while F_0_ decreased significantly in the MD compared to the MD-1 period. This pattern indicates higher theoretical maximal velocity (V_0_) alongside lower F_0_.

Aligned with the results presented in this study, previous investigations [27] revealed slight, non-significant improvements in CMJ height following an individualized strength session performed the day before competition in football players. Similarly, other studies [7] demonstrated non-significant improvements in CMJ compared to baseline. This improvement could be attributed to the absence of fatigue and the facilitation of explosive performance (short-term tapering effect) [28]. Although our study did not show significant improvements in jump height, there was an increase in jump height over the weeks, indicating that players did not experience a marked drop in performance due to fatigue. However, previous studies [29,30] confirm that periodical comparison of CMJ height is a reliable method for detecting neuromuscular fatigue in athletes.

On the other hand, it has been previously demonstrated [31] that lighter loads are suitable for improving maximal theoretical velocity (V_0_), while heavier loads are more suitable for improving force production at low velocity (F_0_). However, our study found that priming training with heavy loads (80% of body weight) improved V_0_. This may be because the participants were amateur football players, whose fitness levels are far from those of professional players. As amateur footballers, the subjects were not familiar with strength training, which may explain why they improved V_0_ and not F_0_, as untrained subjects tend to improve first in the variable where they have the greatest deficit. Recent research [32] found significant improvements in stronger (more trained) players, but not in weaker (less trained) players, 24 h after priming training. This justifies the absence of significant improvements in the variables studied in our research.

Similarly, it is important to highlight that this study is not without limitations. Football is a multi-component sport, and player performance cannot be adequately represented by isolated, linear tests such as the CMJ or linear sprint. Unlike professionals, amateur players often present greater variability in performance because of uncontrolled contextual factors. These factors—such as lifestyle, nutrition, or occupational activity—were not measured, but they may influence training responses and recovery. This heterogeneity could have influenced individual responses to training. Such variability may limit consistency in performance outcomes. Although a control group was not included, the study aimed to reflect real-world conditions in amateur football, where strict experimental designs are often impractical. This limits causal inference but enhances ecological validity.

Additionally, the time-segmented control-to-intervention design used in this study presents inherent limitations that constrain causal inference. Although this approach allowed for practical implementation within a single squad, it may have introduced confounding factors such as seasonal variation, match congestion, changes in coaching emphasis, and environmental conditions (e.g., weather and pitch quality). These uncontrolled variables could have influenced performance outcomes independently of the intervention. Future studies aiming to better isolate the effects of the intervention may benefit from alternative designs such as parallel control groups, randomized intervention orders, or AB/BA crossover protocols, which could help mitigate temporal and contextual influences.

It is also worth noting that more trained players show greater improvements in priming. This suggests that those with greater experience and skills developed over the years respond better to priming training stimuli. This phenomenon could be due to their greater familiarity with fitness techniques and strategies, as well as a greater ability to adapt quickly to changing conditions.

## 5. Conclusions

This study reveals that sled-resisted training with a heavy load and low volume may not yield immediate improvements in CMJ for amateur football players within 24 h of execution. However, it suggests modest long-term enhancements in CMJ relative force and different changes in sprinting after a consistent 10-week regimen, which should be interpreted with caution.

These findings may offer practical guidance for football coaches and physical trainers, as they suggest potential benefits of this training method for amateur players. The approach, involving just three repetitions per player each week, may contribute to improvements in jumping performance and sprint-related variables. However, these conclusions should be considered preliminary, and further controlled studies are warranted to confirm their applicability. Additionally, the absence of immediate performance gains suggests that this training does not need to be scheduled immediately before competition, allowing greater flexibility in planning.

## Figures and Tables

**Table 1:** Variables CMJ and sprint.

Test	Variable	Definition
	F	Relative force (N/kg)
CMJ	P	Relative power (W/kg)
	Jump height	Maximal height (cm) reached during a jump
Sprint	Time	Total 40 m sprint time (s)
	V_max_	Maximal speed (m/s)
	Peak force	Greatest force (N) recorded during the concentric phase of a movement
	F_0_	Theoretical maximal force (N/kg) (ordinate axis intercept (y) in F-V linear regression)
	V_0_	Theoretical maximal velocity (m/s) (abscissa axis intercept (x) in F-V linear regression)
	P_max_	Maximal power (W/kg)
	RF_max_	Ratio of maximal force (%)
	DRF *	Ability to maintain net horizontal force production despite increasing running speed (%)

* DRF values are expressed as negative percentages, with less negative values (i.e., closer to zero) indicating improved ability to maintain horizontal force as speed increases.

**Table 2:** Linear mixed model for CMJ’s variables results.

Variables		Coefficient	Standard Error	df	*t*-Value	*p*-Value
F (N/kg)	intercept	12.84	0.11	21.06	119.57	0.001
	MD-1–MD = MD	0.01	0.03	450.61	0.30	0.766
	sled = yes	0.10	0.04	434.49	2.44	0.015 *
	MD-1–MD = MD: sled = yes	0.02	0.04	450.61	0.47	0.638
P (W/kg)	intercept	15.85	0.30	19.28	52.57	0.001
	MD-1–MD = MD	0.04	0.12	447.35	0.37	0.710
	sled = yes	0.24	0.14	281.30	1.74	0.082
	MD-1–MD = MD: sled = yes	0.07	0.15	447.35	0.50	0.617
Jump height (cm)	intercept	31.30	0.75	19.97	41.94	0.001
	MD-1–MD = MD	0.13	0.32	442.77	0.41	0.681
	sled = yes	0.45	0.35	199.84	1.30	0.196
	MD-1–MD = MD: sled = yes	0.19	0.39	442.77	0.49	0.627

CMJ variable values. Variables: F = relative force; P = relative power. Intercept = MD-1–MD value MD-1, without sled; MD-1–MD = MD = MD-1–MD value MD (sled = yes and sled = no) minus MD-1–MD value MD-1 (sled = yes and sled = no); sled = yes equal sled = yes minus sled = no; MD-1–MD = MD: sled = yes equal MD-1–MD value MD, with sled minus intercept. * Significant differences (*p* < 0.05).

**Table 3:** Sprint variables: pre–post results (paired *t*-tests).

Variables	PRE		POST		PRE–POST				
	Mean	SD	Mean	SD	MD	95% CI		*p*	ES
						Inf	Sup		
Time (s)	5.73	0.22	5.75	0.29	0.01	−0.08	0.11	0.794	0.05
V_max_ (m/s)	8.73	0.44	8.85	0.48	0.12	−0.1	0.33	0.270	0.25
Peak force (N)	48.58	9.23	50.46	8.82	1.88	−3.25	7.01	0.443	0.21
F_0_ (N/kg)	7.98	0.71	7.67	0.62	−0.31	−0.58	−0.05	0.024 *	−0.47
V_0_ (m/s)	9.05	0.38	9.27	0.48	0.22	0.03	0.4	0.023 *	0.49
P_max_ (W/kg)	18.09	2	17.78	1.82	−0.31	−0.93	0.32	0.306	−0.16
RF_max_ (%)	0.53	0.03	0.52	0.02	−0.01	−0.02	0.00	0.071	−0.34
DRF (%)	−0.08	0.01	−0.07	0.01	0.01	0.00	0.01	0.007 *	0.77

Sprint variable value. V_max_ = maximal speed; F_0_ = theoretical maximal force; V_0_ = theoretical maximal velocity; P_max_ = maximal power; RF_max_ = ratio of maximal force; DRF = decrease in the ratio of horizontal force with increasing speed. SD = standard deviation; MD = mean difference; 95% CI = 95% confidence interval; “Inf” = inferior; “Sup” = superior; *p* = *p*-value; ES = effect size. * Significant differences (*p* < 0.05).

## Data Availability

The datasets generated and analyzed during the current study are available at https://doi.org/10.5281/zenodo.17250211 (accessed on 10 October 2025).

## Abbreviations

The following abbreviations are used in this manuscript:

PAP	Post-activation potentiation
PAPE	Post-activation performance enhancement
CMJ	Countermovement jump
MD	Match day
MD-1	Day before the match
F	Relative force (N/kg)
P	Relative power (W/kg)
V_max_	Maximal speed (m/s)
F_0_	Theoretical maximal force (N/kg) (ordinate axis intercept (y) in F-V linear regression)
V_0_	Theoretical maximal velocity (m/s) (abscissa axis intercept (x) in F-V linear regression)
P_max_	Maximal power (W/kg)
RF_max_	Ratio of maximal force (%)
DRF	Ability to maintain net horizontal force production despite increasing running speed (%)
